# A dosiomics model for prediction of radiation-induced acute skin toxicity in breast cancer patients: machine learning-based study for a closed bore linac

**DOI:** 10.1186/s40001-024-01855-y

**Published:** 2024-05-12

**Authors:** Pegah Saadatmand, Seied Rabi Mahdavi, Alireza Nikoofar, Seyede Zohreh Jazaeri, Fahime Lamei Ramandi, Golbarg Esmaili, Soheil Vejdani

**Affiliations:** 1 Department of Medical Physics, School of Medicine, Iran University of Medical Sciences, Tehran, Iran; 2 Radiation Biology Research Center, Iran University of Medical Sciences, Tehran, Iran; 3 Department of Radiation Oncology, School of Medicine, Iran University of Medical Sciences, Tehran, Iran; 4 Department of Neuroscience, Faculty of Advanced Technologies in Medicine, Iran University of Medical Sciences, Tehran, Iran; 5 Division of NeuroscienceCellular and Molecular Research Center, Iran University of Medical Sciences, Tehran, Iran; 6 Radiotherapy Department, Pars Hospital, Tehran, Iran; 7 Department of Radiation Oncology, Firoozgar Hospital, Iran University of Medical Sciences, Tehran, Iran

**Keywords:** Breast cancer, Radiation therapy, Acute skin toxicity, Machine learning, Dosiomics

## Abstract

**Background:**

Radiation induced acute skin toxicity (AST) is considered as a common side effect of breast radiation therapy. The goal of this study was to design dosiomics-based machine learning (ML) models for prediction of AST, to enable creating optimized treatment plans for high-risk individuals.

**Methods:**

Dosiomics features extracted using Pyradiomics tool (v3.0.1), along with treatment plan-derived dose volume histograms (DVHs), and patient-specific treatment-related (PTR) data of breast cancer patients were used for modeling. Clinical scoring was done using the Common Terminology Criteria for Adverse Events (CTCAE) V4.0 criteria for skin-specific symptoms. The 52 breast cancer patients were grouped into AST 2 + (CTCAE ≥ 2) and AST 2 − (CTCAE < 2) toxicity grades to facilitate AST modeling. They were randomly divided into training (70%) and testing (30%) cohorts. Multiple prediction models were assessed through multivariate analysis, incorporating different combinations of feature groups (dosiomics, DVH, and PTR) individually and collectively. In total, seven unique combinations, along with seven classification algorithms, were considered after feature selection. The performance of each model was evaluated on the test group using the area under the receiver operating characteristic curve (AUC) and f1-score. Accuracy, precision, and recall of each model were also studied. Statistical analysis involved features differences between AST 2 − and AST 2 + groups and cutoff value calculations.

**Results:**

Results showed that 44% of the patients developed AST 2 + after Tomotherapy. The dosiomics (DOS) model, developed using dosiomics features, exhibited a noteworthy improvement in AUC (up to 0.78), when spatial information is preserved in the dose distribution, compared to DVH features (up to 0.71). Furthermore, a baseline ML model created using only PTR features for comparison with DOS models showed the significance of dosiomics in early AST prediction. By employing the Extra Tree (ET) classifiers, the DOS + DVH + PTR model achieved a statistically significant improved performance in terms of AUC (0.83; 95% CI 0.71–0.90), accuracy (0.70), precision (0.74) and sensitivity (0.72) compared to other models.

**Conclusions:**

This study confirmed the benefit of dosiomics-based ML in the prediction of AST. However, the combination of dosiomics, DVH, and PTR yields significant improvement in AST prediction. The results of this study provide the opportunity for timely interventions to prevent the occurrence of radiation induced AST.

**Supplementary Information:**

The online version contains supplementary material available at 10.1186/s40001-024-01855-y.

## Background

Among all the vital constituents of breast cancer treatment, adjuvant radiotherapy (RT) plays a paramount role in substantially enhancing overall survival rate and effectively reducing risk of localized cancer recurrence [1, 2]. Recent developments in RT techniques have shown that Tomotherapy can be employed to achieve optimal target coverage through meticulous dose painting, which results in improving tumor control probability and reducing normal tissue complications in comparison with traditional techniques [3–5]. Nonetheless, considering patient’s quality of life and undesirable treatment interruptions, acute skin toxicity (AST) remains a notable concern in breast cancer RT [6]. Thus, through timely prediction of skin complications, there might be a possibility to reduce skin toxicity and provide biologically optimized treatment plans for breast patients.

The role of treatment plan physical quantities including skin dose distribution and dose volume histograms (DVHs) [7, 8], as well as patient’s demographic and clinical characteristics have been investigated in predicting AST in breast RT by several investigators [9, 10]. However, DVHs lack spatial information of the dose of treatment plans [11]. A recent literature review of 38 studies has shown considerable heterogeneity in assessing acute radiation dermatitis and contributing risk factors [12].

On the other hand, previous investigations have revealed limited performance of statistical and radiobiological based models, i.e., normal tissue complication probability (NTCP) models, in predicting individual patients' skin toxicity [13, 14]. Nevertheless, by extracting spatial information from dose distribution using dosiomics, i.e., a texture analysis (TA) approach, prediction capability can be significantly improved [15]. Despite numerous studies emphasizing the improved predictive capability of dosiomics in assessing toxicity following RT, skin toxicity has not been evaluated in those investigations [15–17].

Recent studies have highlighted the effectiveness of machine learning (ML) to construct predictive models for skin toxicities by incorporating patient and treatment-related features either individually [13], or in combination with quantitative thermal imaging biomarkers (i.e., thermoradiomics) [18], spectrophotometric markers [19], and planning CT image [20]. Nonetheless, the utilization of additional imaging devices in these studies has some limitations, including high implementation and maintenance costs, which indirectly affect patient expenses. Moreover, this might increase clinic workload, and patient discomfort [18, 20]. The limitation of planning CT images in predicting skin toxicity lies in its reliance on anatomical features, failing to present specific predictions for different treatment plans [21].

To the best of our knowledge, this research represents a novel method to predict AST using a combination of dosiomics, DVH and patient-specific treatment-related (PTR) features for ML modeling.

## Methods

### Study design

**Fig. 1:**
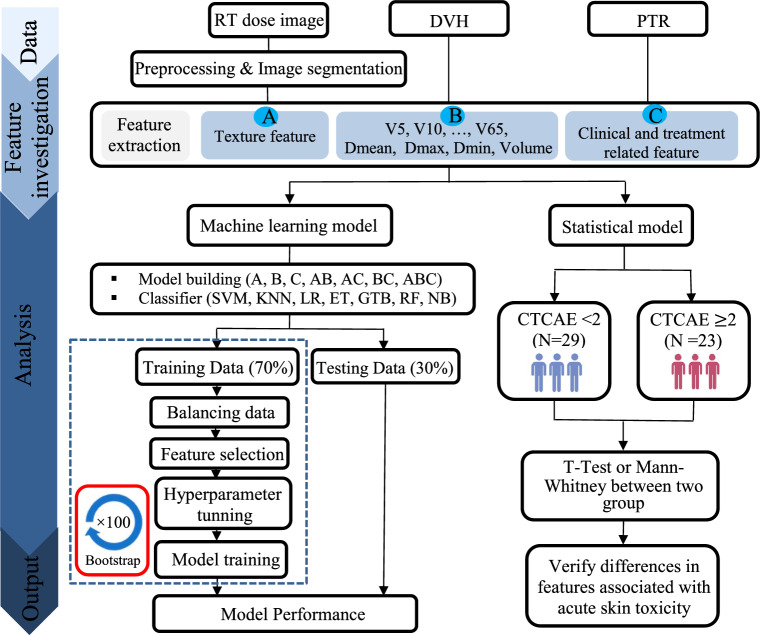
Flow chart of this study

This study included a prospective analysis of 52 patients with confirmed invasive breast carcinoma who received post-operative Tomotherapy at our center from 2020 to 2023. The study protocol was approved by the institutional Ethics Committee (IR.IUMS.FMD.REC.1400.591). Patients were excluded if they had a history of collagen vascular disease, prior breast/thoracic radiation therapy, or were received topical corticosteroids/antibiotics. Fig1 illustrates the flow chart followed in the present study.

### Patients

Patient’s computed tomography (CT) simulation (Siemens SOMATOM 64-slice) was performed, using 2 mm slice thickness, in the supine position with arms raised above the head. Then, images were transferred to Tomotherapy treatment planning system (TPS), Precision, (Accuray, Inc., Sunnyvale, CA). Using a threshold density of 0.55 g/cm^3^, the TPS automatically detected and generated the body surface. After segmenting clinical target volumes (CTV) and adjacent organs at risk (OARs), according to the ICRU reports 50, 62, and 83, planning target volumes (PTV) were generated by adding 5 mm margins in all directions to the CTV. Moreover, to collect skin dosimetric data from the planning CT scan, an automatic 2 mm strip from the chest area’s body surface (skin layer 2 ([SL2]) was generated in the TPS. The SL2 layer was created by limiting a 2-mm body surface strip in the thoracic region to the adjacent areas of the PTV, extending craniocaudally corresponding to the PTVs on the planning CT [8].

Patients received 6 MV X-ray treatments in a conventional (50 Gy in 25 fractions) or hypofractionated schedule (42.4 Gy in 16 fractions) with an optional tumor bed boost (10 Gy in 5 fractions), to cover at least 95% of the PTV volume. The OAR planning objectives, guided by QUANTEC recommendations [22] and additional acceptable limits [23, 24], were as follows: V_5_ < 50%, V_20_ < 20% (V_x_ defined as the percentage of the total volume exceeding x Gy) with mean dose 15 Gy for ipsilateral lung, mean dose of 2 Gy for Contralateral Lung, V_5_ ≤ 15.0% with mean dose of 2 Gy for Contralateral breast, V_25_ < 10% with mean dose of 4 Gy for heart, maximal dose of 10 Gy for esophagus, mean dose of 20 Gy for thyroid gland and maximal doses of 45 Gy for spinal cord. The skin dose was minimized by clipping all PTVs from the body surface by 3 mm [25, 26]. The planning requirements did not involve dose constraints for skin, but skin dose was monitored to be kept as low as possible without compromising target volume coverage. The dose distribution was calculated using a high spatial resolution mode in TPS, matching the imported CT data resolution. Patient and treatment-related features (Table 1), including; DICOM RT plan, RT dose, and RT structure were collected to create the ML models.

**Table 1:** Patient clinical and treatment characteristics

Characteristic	Mean (range)/n (%)
Age (years)	50 (34–81)
BMI	24.49 (19.83–45.71)
Target volume	964.9 (421.78–3766.31)
Tumor location	
Right sided	17 (33%)
Left sided	35 (67%)
TNM stage	
1	15 (29%)
2	25 (48%)
3	10 (19%)
4	2 (4%)
Chemotherapy	
Yes	44 (85%)
No	8 (15%)
Fraction schedule	
Conventional	37 (71%)
Hypofractionation	15 (29%)
Treatment area	
Breast	21 (40%)
Breast with lymph node	22 (42%)
Chest wall	1 (2%)
Chest wall with lymph node	8 (15%)
Boost	
Yes	39 (75%)
No	13 (25%)
Surgery type	
Mastectomy	10 (19%)
Lumpectomy	42 (81%)
Skin toxicity grade	
< 2	29 (56%)
≥ 2	23 (44%)

### Dose–volume histogram features

The dose–volume histogram (DVH) features for patients in the analyses were consisted of 30 dosimetric parameters including the SL2 receiving x Gy or higher (Vx) in both percentage and absolute volumes [cc], with x ranging from 5 to 65 Gy in 5 Gy steps, and also total volume, maximum (D_max_), minimum (D_min_), and mean (D_mean_) of SL2.

### Dosiomics feature

Utilizing the Pyradiomics tool (v3.0.1), dosiomics features were obtained following the resampling of dose distributions to 1 × 1 × 1 mm^3^ using the b-spline algorithm and discretization of gray level intensity into fixed bins of 1 Gy.

By analyzing the SL2 segment, a comprehensive set of 107 dosiomics features was derived, encompassing shape, gray level co-occurrence matrix (GLCM), gray level dependence matrix (GLDM), gray level run length matrix (GLRLM), gray level size zone matrix (GLSZM), neighboring gray-tone difference matrix (NGTDM), and gray level histogram as first-order features.

### End point

For each patient, grades of radiation-induced skin toxicity were recorded by assigning the maximum score observed via visual inspection, in weekly follow-ups during the treatment and within 3-month post-treatment. Common Terminology Criteria for Adverse Events (CTCAE) version 4.0 was used for scoring [27], which has previously been utilized in breast cancer radiotherapy [12]. Patients were divided into 2 groups of AST 2 + (CTCAE ≥ 2) and AST 2- (CTCAE < 2) for modeling.

### Modelling

#### Univariate analysis

To start, features were undergone z-score normalization for zero mean and unit variance. Each feature was assessed using univariate logistic regression analysis to determine its predictive value, including; the area under the receiver operating characteristic curve (AUC) measure and its statistical associations with AST 2 +. Using bootstrap resampling of the original dataset, the univariate regression model was iteratively built 100 times. The average AUC of the bootstrap samples was used as a measure of its predicted performance. The statistical significance was demonstrated by a Bonferroni-corrected *p* value below 0.05/150 ≅ 0.0003. Notably, feature selection did not utilize the univariate analysis.

#### Multivariate analysis

Multivariate analysis encompasses multiple steps, including feature scaling, feature selection, class balancing, and classification. Fig 1 illustrates an overview of the ML model followed in the present study. The z-score normalization scaled all characteristics for zero mean and unit variance. Patients were randomly split into training (70%) and testing (30%) cohorts, enabling the evaluation of models on unseen data. To improve the trained classifier's performance in generalization on the unseen data and overcome the limitations of the limited dataset, 100 bootstrap samples (with replacement) are generated through bootstrap resampling of the training data [15, 18].

To rectify the discrepancy in observed case frequencies between the two classes, the training set was utilized the synthetic minority oversampling technique (SMOTE), to randomly oversample the minority class. It should be noted that repeated application of SMOTE during bootstrap resampling aimed to minimize biased results. In each bootstrap iteration a two steps feature selection was conducted: First utilizing Spearman's correlation analysis, redundant features were eliminated by removing one of the two features that exhibit a strong correlation coefficient (CC) with the remaining features following the presence of a high CC between the two features (CC ≥ 0.8). Then, important features were selected after fitting the Extra-Trees (ET) classifier as the base models on the remaining feature sets. After fitting, the ET algorithm assigns importance scores to the features, and subsequently, the least-ranked features are eliminated from the feature set [28, 29]. This process was repeated for each feature set (PTR, DVH, dosiomics) individually or in combination to identify the top 10 significant features for each model. Feature selection with the Extra Trees Classifier efficiently manages noisy, high-dimensional data, simultaneously reducing bias. Selected features were utilized to train seven supervised classifier algorithms, including; support vector machine (SVM), k-nearest neighbors (KNN), logistic regression (LR), gradient tree boosting (GTB), random forest (RF), Naive Bayes (NB), and ET. By implementing a random search within an inner cross-validation loop, the hyperparameters of each model were adjusted to discover the optimal hyperparameter configurations that yielded superior performance for the model. Additional file 1 contains comprehensive hyperparameter details for the seven classifiers. Fitted ML models on each bootstrap sample were tested on unseen data through bootstrapping with 100 repetitions. In the end, multiple prediction models were examined using multivariate analysis, utilizing various combinations of feature groups, including dosiomics (DOS), DVH, and PTR features individually as well as in combination, resulting in 7 distinct combinations in total.

### Performance evaluation

This research employs commonly accepted binary classification metrics to assess the model’s probability assignments [30, 31], which are guided by domain knowledge and previous relevant studies [32, 33]. The performance of each model on the test data was evaluated using the average, and 95% confidence interval (CI) AUC through the bootstrapping approach. The accuracy, precision, recall, and f1-score, were estimated.

The AUC score assesses how effectively a classifier can separate positive and negative classes based on its predicted probabilities. We calculated recall ($\frac{{\text{TP}}}{{\text{FN}}+{\text{TP}}}$) to assess the classifier’s ability to identify positive labels effectively, precision $\left(\frac{{\text{TP}}}{{\text{TP}}+{\text{FP}}}\right)$ to measure concordance of data labels and positive classifier labels, overall accuracy ($\frac{{\text{TP}}+{\text{TN}}}{{\text{TP}}+{\text{FP}}+{\text{TN}}+{\text{FN}}}$) to assess the classifier’s overall performance, and the f1 score ($\frac{2\mathrm{ TP}}{2\mathrm{ TP}+\mathrm{ FN}+{\text{FP}}}$) as a metric that combines precision and sensitivity into one value, where TP stands for true positive, TN for true negative, FP for false positive, and FN for false negative [31]. In medical applications, recall is crucial when missing positives is costly as it ensures that no actual cases of high-risk patients are overlooked. Precision is essential when costly false positives can occur, as it averts mislabeling low-risk patients as high-risk, saving time and money. The F1 score ensures a balanced evaluation of a model's ability to identify both patient groups effectively. However, accuracy evaluates the total performance of predictions across both groups [30]. Statistical analysis using Python and SciPy packages was involved conducting *Z* tests to compare the statistical significance of AUC values between pairs of models with a significance level of *p* value < 0.05. The challenge of multiple comparisons necessitated the application of the Bonferroni correction method to control the error rate. By adjusting the significance level, achieved by dividing 0.05 by the total number of comparisons (49 ML models for comparison of 7 prediction models using 7 classification algorithms), the error rate is effectively controlled.

### Statistical analysis

SPSS Version 27 (IBM Corp, Armonk, NY) was used to compare mean continuous variables between AST 2 − and AST 2 + groups, applying independent samples *t* test or Mann–Whitney tests, depending on data distribution normality [34]. Categorical variables between the two groups were compared using a Fisher exact test. In this study, *p* values < 0.05 were considered as significant level. Ultimately, the optimal cut-off point for selected features in the best prediction model was determined by maximizing Youden's index on the ROC curve [35].

## Results

Among the 52 patients who participated in this study, 44% experienced AST 2 + complication during the 3-month follow-up period. The demographic and clinical characteristics are illustrated in Table 1. Table 2 presents the results of the univariate analysis, highlighting the AUC and odds ratio (OR) for the top ten features, which were identified as high correlation with AST 2 +. This table indicates a significant statistical association between AST 2 + and the features of the maximum gray level histogram (OR = 3.23), range of the gray level histogram (OR = 3.79), as well as the fraction schedule (OR = 1.24). Among the DOS, DVH, and PTR groups, the maximum gray level histogram (AUC = 0.87), V_55Gy_ (cc) (AUC = 0.78), and fraction schedule (AUC = 0.70) features demonstrated the highest AUC values.

**Table 2:** Univariate analysis findings

Feature group	Feature name	OR		AUC		*p* value	
		Median	10th − 90th%	Median	10th − 90th%	0.05	0.0003
DOS	First order (Maximum)	3.23	1.16–8.89	0.87	0.73–0.96	*	*
	First order (Range)	3.79	1.43–8.44	0.87	0.71–0.97	*	*
	First order (Entropy)	1.86	0.73–4.37	0.79	0.64–0.94	*	NS
	GLCM (Sum Entropy)	2.09	0.82–4.66	0.80	0.62–0.95	*	NS
	GLCM (Difference Variance)	4.50	0.96–21.85	0.77	0.61–0.92	*	NS
	GLCM (Joint Entropy)	2.62	0.86–8.06	0.76	0.57–0.92	*	NS
	GLSZM (Size Zone Non Uniformity)	1.76	0.69–7.22	0.75	0.59–0.90	*	NS
DVH	V_55Gy_ (cc)	1.62	0.49–16.37	0.78	0.64–0.89	*	NS
	D_max_	1.56	0.69–4.01	0.77	0.61–0.90	*	NS
PTR	Fraction schedule	1.24	0.54–2.38	0.70	0.57–0.82	*	*

The *p* values indicate the statistical significance of the associations between each feature and AST: a standard *p* value of 0.05 and a Bonferroni-corrected *p* value of 0.0003 are applied

OR = odds ratio; area under the receiver operating characteristic curve (AUC); NS = nonsignificant; *: Significant

**Fig. 2:**
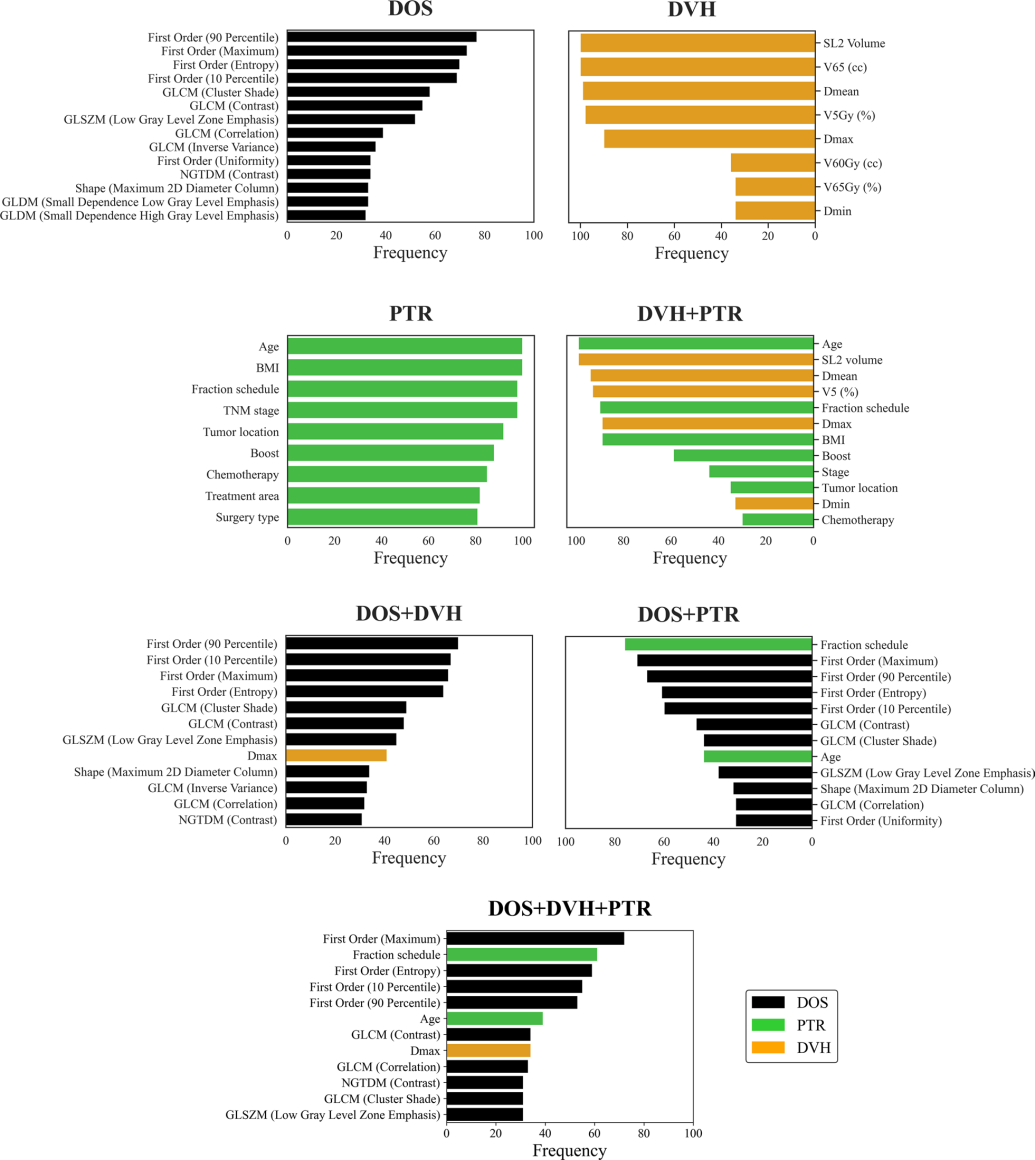
Notable features of each model from 100 bootstrap runs, with a focus on features that were chosen more than 30 times (30%)

Features that were chosen in over 30% of each model from 100 bootstrap runs are ranked in Fig. 2. Among all the selected features, the 90th percentile of the gray level histogram, volume SL2, and age emerged as the most frequent features chosen from the DOS, DVH, and PTR models, respectively. When considering all groups together, the maximum gray level histogram, fraction schedule, and D_max_ of the SL2 segment was revealed as the most frequently selected feature from each group during the feature selection process.

**Fig. 3:**
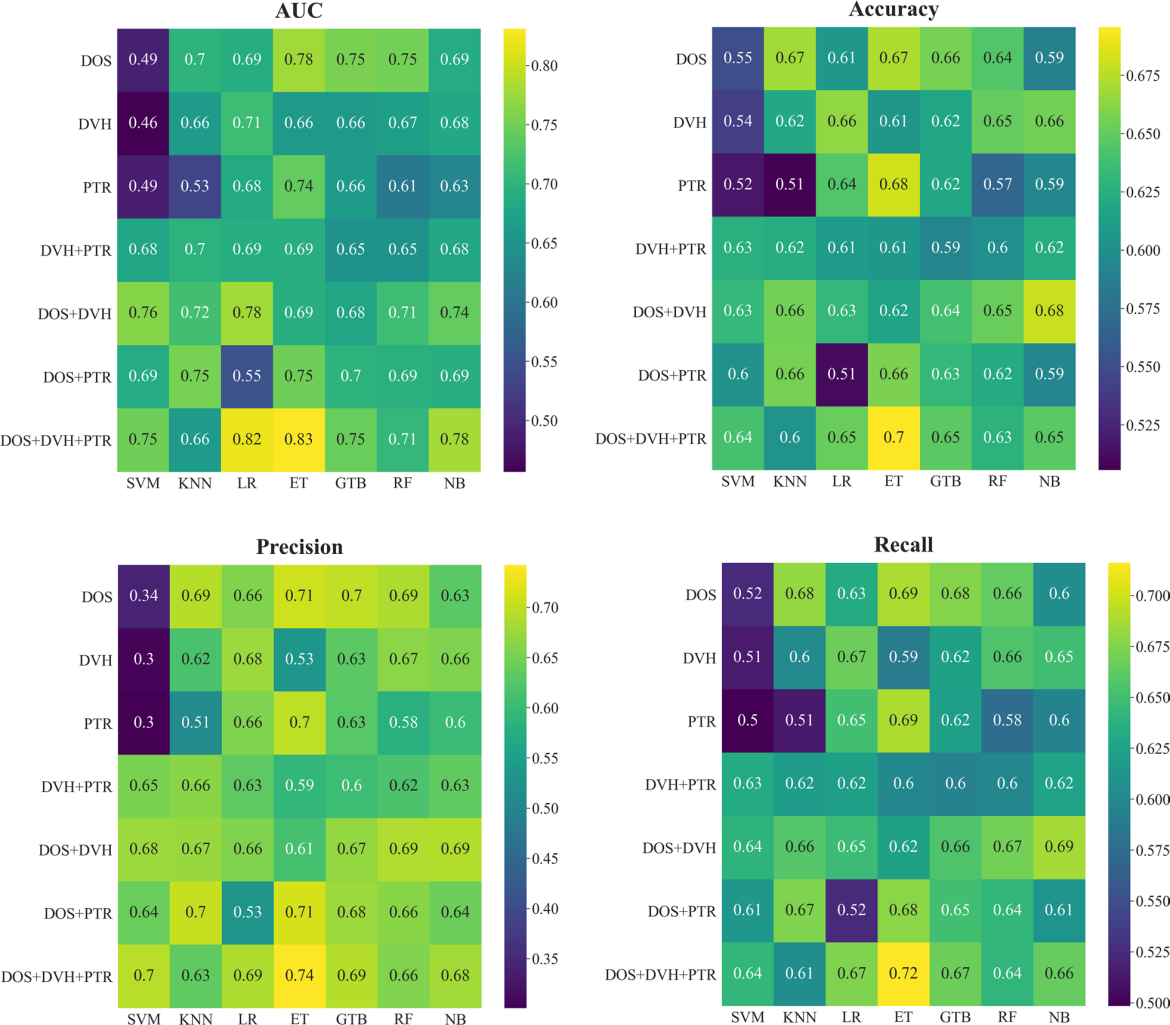
Comparison of prediction performance for different models and classification algorithms

**Fig. 4:**
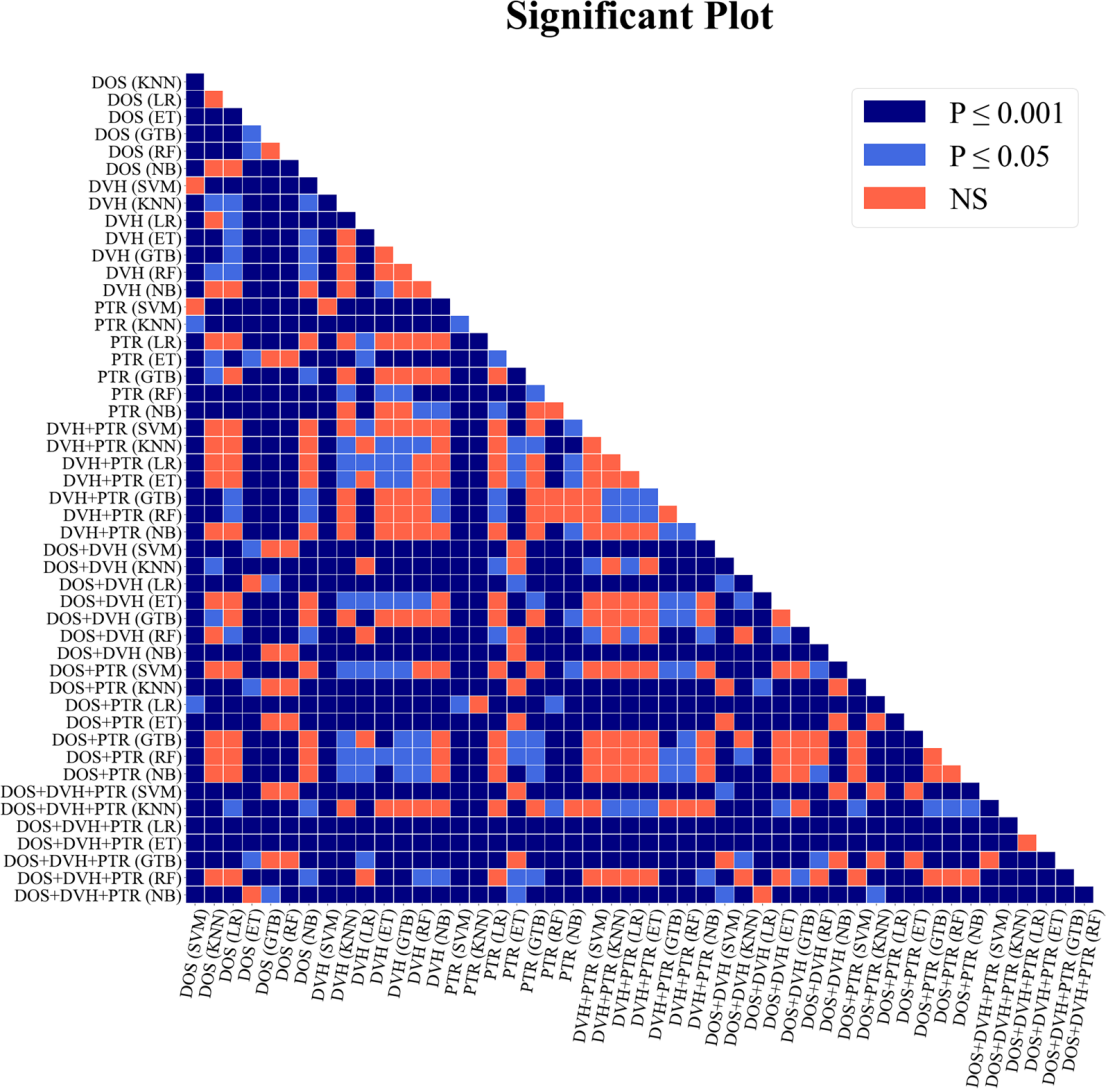
Statistical significance comparison across all model pairs. P indicates the level of significance for the differences in AUCs between each pair of models: 0.05 denotes the typical threshold for significance, and 0.001 represents the significance level adjusted using the Bonferroni method. NS = nonsignificant

Performance of the model is summarized in Fig. 3, showing the results in terms of AUC, accuracy, precision, and recall. These results are extensively documented in Additional file 1: Table S1. Using a *Z* test, the statistical significance between the AUC values of every pair of models is depicted in Fig. 4. Through the evaluation of models using AUC, it was determined that the Extra Tree (ET) and Logistic Regression (LR) machine learning algorithms resulted in models with higher performance. By employing the ET and LR classifiers, the DOS + DVH + PTR model achieved a statistically significant improvement (*p* < 0.001) in AUC values, with values of 0.83 (95% CI 0.71–0.90) and 0.82 (95% CI 0.74–0.89), respectively.

However, ET classification algorithms demonstrated the best performance in terms of accuracy (0.70 ± 0.01), precision (0.74 ± 0.06), and sensitivity (0.72 ± 0.01) for the DOS + DVH + PTR model. Additional file 1: Fig S1 includes ROC curves for seven models on test data, which are plotted per classifier.

**Fig. 5:**
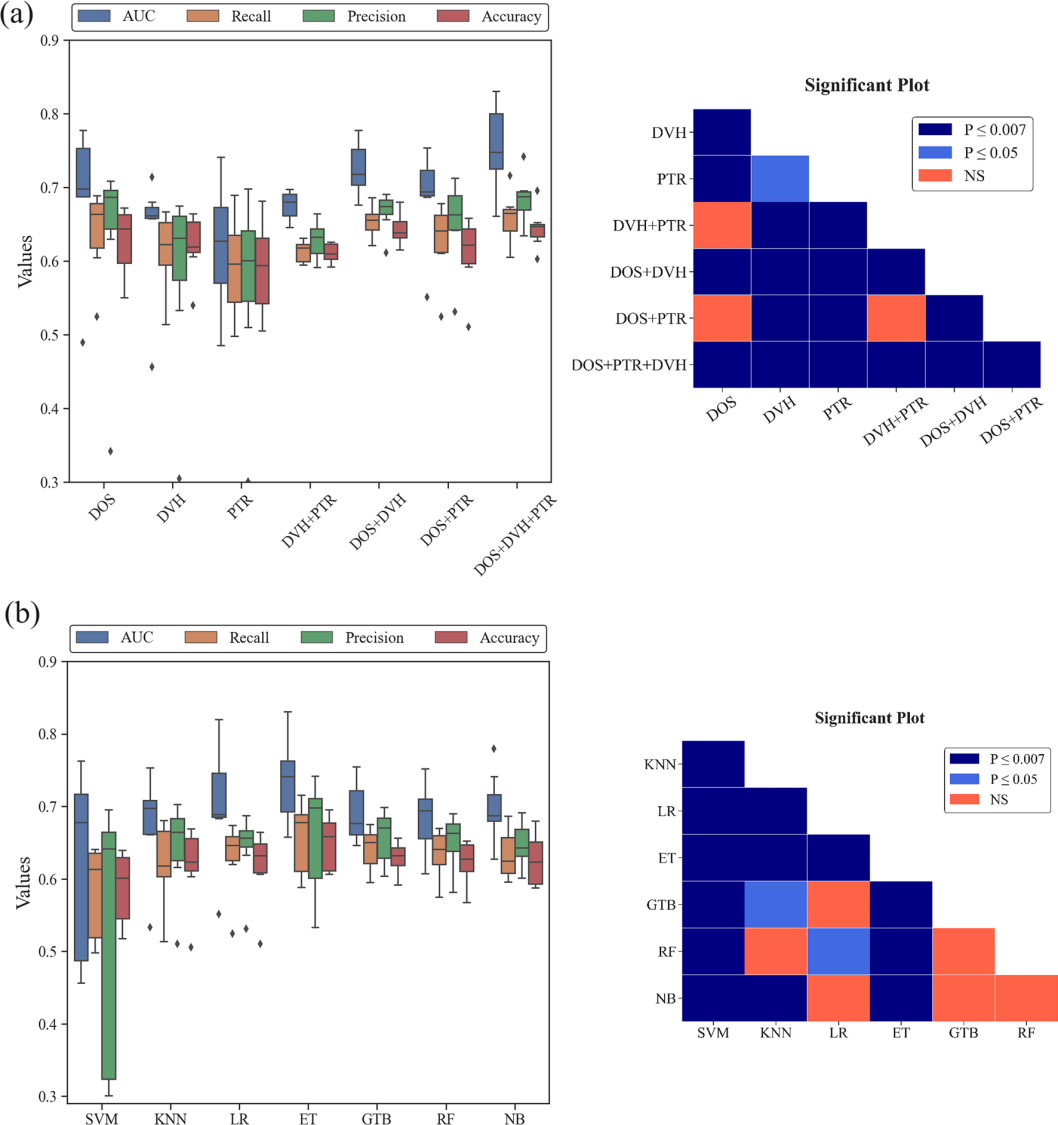
Boxplots for comparison of the performance of **a** various ML models under 7 classifiers and **b** various classification algorithms under 7 ML models used in this study, along with a statistical significance comparison of the average AUC. P indicates the level of significance for the differences in AUCs between models or classifiers: 0.05 denotes the typical threshold for significance, and 0.007 represents the significance level adjusted using the Bonferroni method. NS = nonsignificant

Moreover, the box plot in Fig. 5 depicted the performance variations among the ML models and classifiers utilized in this research, presenting statistically significant results (Bonferroni corrected *p* value < 0.007) for the average AUC. Based on statistical analysis, the DOS + DVH + PTR and PTR models were respectively recognized as the best (average AUC value of 0.76) and worst (average AUC value of 0.62) models in terms of performance among all ML models.

Furthermore, among all the classifiers, the ET and SVM classifiers displayed the strongest and weakest performance, respectively, as evidenced by an average AUC value of 0.73 and 0.62. The DOS model provided a significantly higher AUC (*p* < 0.007) compared to the DVH model. Additional file 1: Table S2 provides a summary of the features that demonstrate a significant association with AST 2 + as determined by a two-tailed *t* test. The optimal cut-off point for selected features in the best prediction model is illustrated in Table 3.

**Table 3:** Result of cut-off value for the final features list

Feature group	Features	Cut-off value	*p* value	Std. error	AUROC	95% CI	Youden index J
DOS	First order (Maximum)	> 55	< 0.0001	0.055	0.838	0.710–0.926	0.576
	First order (Entropy)	> 5.198	< 0.0001	0.067	0.778	0.641–0.882	0.507
	First order (10 percentile)	≤ 11	0.173	0.083	0.614	0.469–0.746	0.394
	First order (90 percentile)	> 50	0.242	0.085	0.599	0.454–0.732	0.297
	GLCM (Contrast)	> 13.93	0.018	0.078	0.685	0.542–0.807	0.409
	GLCM (Correlation)	≤ 0.915	0.255	0.086	0.598	0.453–0.732	0.400
	NGTDM (Contrast)	> 0.206	0.078	0.083	0.646	0.501–0.774	0.375
	GLCM (Cluster Shade)	> − 5194.66	0.806	0.088	0.522	0.379–0.662	0.270
	GLSZM (Low Gray Level Zone Emphasis)	> 0.005	0.889	0.091	0.513	0.370–0.654	0.304
DVH	D_max_	> 54.87	< 0.0001	0.066	0.759	0.620–0.866	0.445
PTR	Fraction Schedule	Hypofractionation	< 0.0001	0.052	0.720	0.578–0.835	0.439
	Age	> 45	0.124	0.079	0.621	0.476–0.752	0.231

## Discussion

Dosiomics-based ML methods demonstrate encouraging prospects in the prediction of treatment toxicity in RT [15–17]. This study utilized dosiomics features extracted from 3D dose distribution of patients’ skin to establish an AST 2 + prediction model for breast cancer patients who have received Tomotherapy. The development of pre-treatment AST 2 + predictive models offers an opportunity for early identification and timely management of high-risk individuals, allowing for the treatment plans optimization, restricting the risk of subsequent damage, and ultimately, enhancing patients’ satisfaction and well-being.

Meticulous dose painting of Tomotherapy in combination with Image Guidance Radiation Therapy (IGRT) and Adaptive Radiation Therapy (ART) [8] can potentially reduce patient-specific variations among planned and delivered doses, impacting the accuracy of predictive models [36].

It has been shown that dosiomics features, along with the preservation of spatial information in the 3D dose distribution, offer superior predictive capability compared to conventional DVH methods [15].Wu et al. developed a multi-stacking deep learning framework for the prediction of radiation-induced dermatitis (CTCAE grade ≥ 2) of patients who underwent breast cancer radiotherapy incorporating multi-region dose-gradient-related radiomics features extracted from pre-treatment planning four-dimensional-CT images, with clinical and dosimetric features. They attained AUCs of 0.82, 0.82, 0.77, 0.80, and 0.80 in the verification data set using RF, XGBoost, AdaBoost, GBDT, and LGBM models as base learners, respectively, which is comparable to the results of this study. However, the GB meta-learner was the best multi-level stacking ensemble method to predict radiation-induced dermatitis 2 + with an AUC of 0.97 in the training dataset and 0.93 in the validation dataset [37]. Feng et al. developed an ML tool with the highest AUC of 0.911 [95% CI 0.838–0.983] through GBDT modeling utilizing internal cross-validation to predict the incidence of radiation dermatitis ≥ 2 using radiomics features derived from multiple dose-gradient-based ROIs of patients' planning CT images, in conjunction with clinical and dosimetric parameters [38]. However, further verification is needed in another center [38].

Furthermore, incorporating multiple ROIs in the radiomics workflow introduces complexity, necessitating extra efforts for segmentation, feature extraction, and subsequent analysis, which can be time-consuming and resource-intensive, particularly for large datasets, and further also potentially increasing the intricacy of interpretation and clinical relevance. In comparison with radiomics, which relies on anatomical and metabolic features, dosiomics provides specific information about the association between radiation dose distribution and treatment outcomes, allowing for unique predictions even when dealing with identical data but different treatment plans [21]. Although relatively few studies emphasize the improved predictive capability of dosiomics in combination with radiomics features in assessing toxicity following RT [39, 40], skin toxicity has not been evaluated in those investigations. Therefore, additional research should be conducted to determine the efficacy of integrating dosiomics and radiomics in enhancing the ability to predict radiation-induced skin toxicity.

Using thermal characteristics of the fifth treatment fraction, Saednia et al. constructed a ML predictive model and predicted CTCAE grade ≥ 2 skin toxicity at the end of breast RT with an AUC of 0.98 and accuracy of 0.87 [18].

Furthermore, limited information on 2D surface thermal imaging constrains the model's predictive performance and the optimization guidance for 3D dose distribution [18].

A recent study by Cilla et al. introduced an ML model to predict severe skin toxicity by incorporating spectrophotometric markers and clinical features. They observed that employing the RBF kernel in the SVM classifier resulted in the best performance, with an accuracy of 89.8% [19]. The classification and regression tree (CART) model identified patients with higher breast volume and melanin index ≥ 99 as correlated with Radiation Therapy Oncology Group (RTOG) grade ≥ 2 skin toxicity with an AUC = 0.959 [19]. Nonetheless, external validation with separate data sets is required to generalize the model developed in this study to new and diverse patients. In addition, dosimics-based prediction can lead to reduced patient costs, clinic workload, and increased patient comfort and tolerance, compared to prediction using additional imaging devices like thermal imaging [18], spectrophotometry [19], and laser Doppler flowmetry [41].

Accurate dose calculation in the 3D dose distribution from Tomotherapy TPS yields precise superficial doses (< 5% error) [42, 43] and ensures that dosiomics features truly represent the delivered radiation dose. Among the participants in this study, 44% experienced AST 2 +, which was in good agreement with the prevalence range documented in previous works [12, 44]. Despite the mild difference between classes, SMOTE was utilized to address the class imbalance issue resulting from high-dimensional data's bias toward the majority class. Oversampling improves the model’s ability to identify and classify positive cases when missing positives are costly.

Consistent with prior research findings [45, 46], this study demonstrates a positive correlation between skin dose and the likelihood of radiation dermatitis.

Previous research suggests that a 2 mm skin layer outperforms 3 mm and 5 mm rings for dosimetric prediction of severe radiation dermatitis in the head and neck region [47]. However, acute radiation-induced skin damage is primarily caused by changes in the epidermis [48, 49] and the papillary dermal vasculature that supplies the epidermis [48, 50]. Regarding the total thickness of the epidermis (76.9 µm) [51] and papillary dermis (60–120 µm) [52] in breast skin less than 2 mm, a 2 mm skin layer is sufficient to assess AST in breast cancer.

In agreement with previous studies [53, 54], our findings indicated that maintenance of spatial dose information results in a significant improvement (*p* < 0.007) in the AUC of dosiomics features (up to 0.78) compared to DVH features (up to 0.71). Our results showed that the ET classifier achieved better performance than other models. Furthermore, combining DVH, dosiomics, and PTR features offered better predictive performance compared to using them individually, suggesting the value of integrating various data sources to enhance prediction accuracy [36]. However, Feng et al. found that adding clinical and dosimetric parameters to radiomics characteristics did not enhance the performance of the prediction model for radiation dermatitis following breast irradiation [38]. Furthermore, physiological cytokines might give useful information in addition to dosimetric data for predicting toxicity outcomes [55]. Complex multi-omics interactions between biological, imaging, and physical data can improve outcome prediction [55].The efficacy of fraction schedules such as hypofractionation [56, 57] and moderate hypo-fractionation as well as its association with the risk of radiation dermatitis has been previously studied [58]. This study corroborates the association between fraction schedule and AST 2 + [12] since acute toxicity is mainly influenced by the total dose [59].

The ML model developed with PTR features served as a baseline for comparison with the dosiomics feature-based model. The AUC of 0.74 achieved by the PTR model in this research closely resembled the 0.77 AUC, as reported in the validation dataset by Aldraimli et al.

In the REQUITE multicenter study, they aimed to predict acute breast desquamation following whole breast external beam radiation therapy, considering demographic and treatment-related characteristics [13]. Nevertheless, Rattay et al. assessed models during external validation incorporating patient and treatment-related factors following breast radiotherapy, obtaining an AUC of 0.65 for acute erythema but failing to predict acute desquamation [60]. Whereas in this study, the outcome of the *Z*-test comparison between the DOS model and the baseline model highlighted the substantial predictive power of dosiomics features. This indicates that textural information of 3D dose distribution plays a crucial role in predicting skin toxicity following radiation treatment.

In this study, we employed a diverse set of common classifiers to predict AST in radiotherapy. These algorithms were selected for their binary classification capabilities and past prediction achievements in radiotherapy [33]. The SVM classifier, with lower overfitting risk, can be influenced by noisy or irrelevant features, resulting in significant AUC variation (0.46–0.76) across groups. Nonetheless, ET, an ensemble of decision trees, offers robustness against noisy features and excels in datasets with a high number of features. Despite LR’s good performance in small datasets, yielding comparable AUC (up to 0.82) with the ET classifier, it might overstate accuracy due to sampling bias. Moreover, KNN utilizes a simple algorithm, but it grants equal importance to features. The RF classifier’s ability to handle large datasets is notable, yet its results show an acceptable AUC (up to 0.75). RF and NB show an acceptable performance in the results, although it scales well for large datasets. Despite the considerable success of the GTB classifier, it can be sensitive to overfitting when applied to noisy data, thereby complicating the tuning process [61, 62]. While traditional ML methods such as SVM, RF, Bayesian networks (BNs), and neural networks (NNs) exhibit promise in multi-omics outcome modeling, several reviews [33, 55] underline their limitations, advocate for deep learning's capacity to surpass current barriers [55]. Deep learning neural network architectures can encompass a larger and heterogeneous set of features, eliminating the requirement for feature selection in conventional models, and incorporating time-to-event data for multi-endpoint prediction [55]. Future research will benefit from the application of a deep-learning neural network that directly receives 3D dose distribution for AST prediction.

In the independent sample *t* test, among the features that showed a significant difference between patients’ groups, D_max_, fraction schedule, the contrast of NGTDM, the contrast, and correlation of GLCM features were identified as selected features with a frequency greater than 30% from the DOS + DVH + PTR model. It is worth mentioning that, higher values of GLCM contrast, a measure of the local variety of the dose distribution, along with higher NGTDM contrast, a measure of the dynamic range of the dose distribution and the extent of local dose distribution variation, and higher D_max_, a measure of maximum dose received by SL2 were associated with AST 2 +. Conversely, lower values of GLCM Correlation, a measurement of Correlation among the dose received by neighboring pixels, were associated with AST 2 +. NGTDM contrast helps in characterizing the local heterogeneity of the radiation dose within the skin, and GLCM contrast and correlation features play a role in identifying regions with steep dose gradients and evaluating the uniformity of dose distribution. The clinical relevance of this study consists of identifying optimum cut-off values for 12 features that were selected in the best dosiomics prediction model with statistical significance. The suggested maximum skin dose to lower the risk of AST following breast radiotherapy was 54.87 Gy, which closely aligns with the cut-off value of 55 for first-order maximum feature, representing the maximum gray level intensity in the skin.

Alongside the usual dose volume limitations, the incorporation of quantitative skin information into dosiomics-based optimization, adhering to the specified thresholds in Table 3, offers the possibility of reducing the incidence of AST after breast radiotherapy.

Variability in dosiomics features, arising from factors including radiotherapy techniques, treatment planning system, grid resolutions, and dose calculation algorithms might obscure dose–response variations, potentially compromising dosiomics model reliability and result generalization [63, 64]. Adapting a dosiomics model for a different technique may need substantial modification and validation to ensure its accuracy and reliability for that specific application. It is recommended to employ harmonization strategies to address potential dose distribution discrepancies caused by variances in radiation treatments [63].

Although our models demonstrated promising predictive ability for AST 2 +, this study has several limitations. First, the sample size is relatively small, potentially limiting the generalizability of the results. Nevertheless, to mitigate the constraint posed by the limited dataset size, bootstrap resampling was employed on the training data.

Second, PTR features show heterogeneity in the sample. However, Regarding the heterogeneity as reported in the fraction schedule [57], chemotherapy [65], surgery type [18], plan boost [46], treatment area [66], and nodal irradiation [67] in similar studies, it appears that these features have a heterogeneous nature. Therefore, considering potential variations in a homogeneous sample size, we recommend a cautious interpretation of the results.

Third, this study focuses on AST, and as such, the predictive ability of the models for chronic skin toxicities remains uncertain. Further research with larger datasets and extended follow-up periods is suggested to confirm and generalize these findings.

In addition, the selection of features may affect the predictive performance. However, we used a rigorous feature selection process and a large number of bootstrapped samples to minimize this bias. Nonetheless, it remains possible that other relevant features not included in our models may improve predictive accuracy [68].

## Conclusion

We successfully established predictive models for AST 2 + in breast cancer patients who received Tomotherapy treatment. The integration of dosiomics, DVH, and PTR features demonstrated enhanced predictive power, allowing for the accurate identification of high-risk patients. The use of ML algorithms, particularly the ET classifier, and multi-modal data fusion represent promising tools for predicting radiation-induced skin toxicity. These models have the potential to facilitate personalized treatment plans, enhance patient satisfaction, and improve the quality of life for breast cancer patients undergoing radiation therapy. However, further investigation with larger datasets and more extended follow-up periods is necessary to validate and generalize our findings.

### Supplementary Information

**Additional file 1:** **Table S1.** Assessing the performance of various machine learning models. **Table S2.** Overview of significant features indicating AST 2+. **Fig S1.** Comparing Model Performance via ROC Curves. **File 1.** The hyper-parameters for the seven classifiers.

## Data Availability

The original contributions presented in the study are included in the article/Additional file. Further inquiries can be directed to the corresponding authors.

## Abbreviations

AST: Acute skin toxicity
DVH: Dose volume histograms
CTCAE: Common terminology criteria for adverse events
RT: Radiotherapy
NTCP: Normal tissue complication probability
TA: Texture analysis
ML: Machine learning
PTR: Patient specific treatment-related factors
TPS: Treatment planning system
CTV: Clinical target volumes
OAR: Organs at risk
PTV: Planning target volumes
SL2: Skin layer 2 mm
D_max_: Maximum dose
D_min_: Minimum dose
D_mean_: Mean dose
GLCM: Gray level co-occurrence matrix
GLDM: Gray level dependence matrix
GLRLM: Gray level run length matrix
GLSZM: Gray level size zone matrix
NGTDM: Neighboring gray-tone difference matrix
AUC: Area under the ROC curve
SMOTE: Synthetic minority oversampling technique
CC: Correlation coefficient
ET: Extra trees
SVM: Support vector machine
KNN: K-nearest neighbors
LR: Logistic regression
GTB: Gradient tree boosting
RF: Random forest
Nb: Naive bayes
OR: Odds ratio
IGRT: Image guidance radiation therapy
ART: Adaptive radiation therapy
