# Supplementary material for: A dosiomics model for prediction of radiation-induced acute skin toxicity in breast cancer patients: machine learning-based study for a closed bore linac
Source: Eur J Med Res. 2024 May 12;29:282. doi: 10.1186/s40001-024-01855-y (PMC11089719; doi:10.1186/s40001-024-01855-y)
Supplement: Supplementary file 1 — Additional file 1: Table S1. Assessing the performance of various machine learning models. Table S2. Overview of significant features indicating AST 2+. Fig S1. Comparing Model Performance via ROC Curves. File 1. The hyper-parameters for the seven classifiers. [file 40001_2024_1855_MOESM1_ESM.docx]

| Supplementary Table 1. Assessing the performance of various machine learning models under 7 classifiers on the test data. | | | | | | | | | | | | | | | | | |
| --- | --- | --- | --- | --- | --- | --- | --- | --- | --- | --- | --- | --- | --- | --- | --- | --- | --- |
| **Model** | **Classifier** |  | **AUC ROC** | | |  | **Accuracy** | |  | **Precision** | |  | **Recall** | |  | **F1 Score** |  |
|  |  |  | Mean | 95% CI | STD |  | Mean | STD |  | Mean | STD |  | Mean | STD |  | Mean | STD |
| DOS | SVM |  | 0.49 | 0.26-0.75 | 0.16 |  | 0.55 | 0.08 |  | 0.34 | 0.19 |  | 0.52 | 0.06 |  | 0.23 | 0.29 |
| DOS | KNN |  | 0.70 | 0.54-0.83 | 0.07 |  | 0.67 | 0.08 |  | 0.69 | 0.08 |  | 0.68 | 0.07 |  | 0.67 | 0.07 |
| DOS | LR |  | 0.69 | 0.57-0.82 | 0.06 |  | 0.61 | 0.06 |  | 0.66 | 0.08 |  | 0.63 | 0.06 |  | 0.65 | 0.06 |
| DOS | ET |  | 0.78 | 0.65-0.88 | 0.05 |  | 0.67 | 0.07 |  | 0.71 | 0.07 |  | 0.69 | 0.06 |  | 0.68 | 0.07 |
| DOS | GTB |  | 0.75 | 0.59-0.90 | 0.08 |  | 0.66 | 0.08 |  | 0.70 | 0.08 |  | 0.68 | 0.08 |  | 0.68 | 0.08 |
| DOS | RF |  | 0.75 | 0.61-0.85 | 0.06 |  | 0.64 | 0.07 |  | 0.69 | 0.07 |  | 0.66 | 0.06 |  | 0.67 | 0.06 |
| DOS | NB |  | 0.69 | 0.60-0.77 | 0.04 |  | 0.59 | 0.05 |  | 0.63 | 0.06 |  | 0.60 | 0.05 |  | 0.60 | 0.08 |
| DVH | SVM |  | 0.46 | 0.22-0.75 | 0.19 |  | 0.54 | 0.07 |  | 0.30 | 0.13 |  | 0.51 | 0.05 |  | 0.20 | 0.29 |
| DVH | KNN |  | 0.66 | 0.48-0.81 | 0.09 |  | 0.62 | 0.08 |  | 0.62 | 0.15 |  | 0.60 | 0.09 |  | 0.46 | 0.23 |
| DVH | LR |  | 0.71 | 0.56-0.78 | 0.06 |  | 0.66 | 0.08 |  | 0.68 | 0.08 |  | 0.67 | 0.09 |  | 0.63 | 0.13 |
| DVH | ET |  | 0.66 | 0.45-0.76 | 0.08 |  | 0.61 | 0.08 |  | 0.53 | 0.20 |  | 0.59 | 0.09 |  | 0.39 | 0.30 |
| DVH | GTB |  | 0.66 | 0.46-0.76 | 0.08 |  | 0.62 | 0.09 |  | 0.63 | 0.10 |  | 0.62 | 0.09 |  | 0.59 | 0.13 |
| DVH | RF |  | 0.67 | 0.45-0.81 | 0.09 |  | 0.65 | 0.09 |  | 0.67 | 0.10 |  | 0.66 | 0.10 |  | 0.62 | 0.15 |
| DVH | NB |  | 0.68 | 0.51-0.75 | 0.07 |  | 0.66 | 0.08 |  | 0.66 | 0.09 |  | 0.65 | 0.09 |  | 0.58 | 0.16 |
| RTP | SVM |  | 0.49 | 0.17-0.83 | 0.16 |  | 0.52 | 0.06 |  | 0.30 | 0.11 |  | 0.50 | 0.04 |  | 0.23 | 0.28 |
| RTP | KNN |  | 0.53 | 0.34-0.8 | 0.12 |  | 0.51 | 0.09 |  | 0.51 | 0.13 |  | 0.51 | 0.09 |  | 0.47 | 0.17 |
| RTP | LR |  | 0.68 | 0.41-0.87 | 0.13 |  | 0.64 | 0.11 |  | 0.66 | 0.11 |  | 0.65 | 0.11 |  | 0.62 | 0.15 |
| RTP | ET |  | 0.74 | 0.45-0.94 | 0.13 |  | 0.68 | 0.10 |  | 0.70 | 0.11 |  | 0.69 | 0.11 |  | 0.66 | 0.15 |
| RTP | GTB |  | 0.66 | 0.46-0.84 | 0.12 |  | 0.62 | 0.12 |  | 0.63 | 0.13 |  | 0.62 | 0.12 |  | 0.58 | 0.14 |
| RTP | RF |  | 0.61 | 0.38-0.86 | 0.15 |  | 0.57 | 0.13 |  | 0.58 | 0.15 |  | 0.58 | 0.13 |  | 0.55 | 0.16 |
| RTP | NB |  | 0.63 | 0.29-0.88 | 0.17 |  | 0.59 | 0.14 |  | 0.60 | 0.16 |  | 0.60 | 0.15 |  | 0.55 | 0.20 |
| DVH+RTP | SVM |  | 0.68 | 0.43-0.86 | 0.13 |  | 0.63 | 0.09 |  | 0.65 | 0.11 |  | 0.63 | 0.10 |  | 0.59 | 0.15 |
| DVH+RTP | KNN |  | 0.70 | 0.48-0.87 | 0.11 |  | 0.62 | 0.09 |  | 0.66 | 0.12 |  | 0.62 | 0.09 |  | 0.54 | 0.17 |
| DVH+RTP | LR |  | 0.69 | 0.46-0.83 | 0.10 |  | 0.61 | 0.09 |  | 0.63 | 0.09 |  | 0.62 | 0.09 |  | 0.60 | 0.11 |
| DVH+RTP | ET |  | 0.69 | 0.42-0.86 | 0.12 |  | 0.61 | 0.09 |  | 0.59 | 0.18 |  | 0.60 | 0.10 |  | 0.47 | 0.27 |
| DVH+RTP | GTB |  | 0.65 | 0.40-0.87 | 0.13 |  | 0.59 | 0.11 |  | 0.60 | 0.13 |  | 0.60 | 0.12 |  | 0.56 | 0.15 |
| DVH+RTP | RF |  | 0.65 | 0.37-0.85 | 0.14 |  | 0.60 | 0.11 |  | 0.62 | 0.13 |  | 0.60 | 0.11 |  | 0.54 | 0.16 |
| DVH+RTP | NB |  | 0.68 | 0.48-0.82 | 0.08 |  | 0.62 | 0.08 |  | 0.63 | 0.09 |  | 0.63 | 0.08 |  | 0.58 | 0.12 |
| DOS+DVH | SVM |  | 0.76 | 0.64-0.84 | 0.05 |  | 0.63 | 0.08 |  | 0.68 | 0.10 |  | 0.64 | 0.07 |  | 0.61 | 0.09 |
| DOS+DVH | KNN |  | 0.72 | 0.61-0.81 | 0.05 |  | 0.66 | 0.06 |  | 0.67 | 0.07 |  | 0.66 | 0.06 |  | 0.62 | 0.10 |
| DOS+DVH | LR |  | 0.78 | 0.69-0.84 | 0.04 |  | 0.63 | 0.07 |  | 0.66 | 0.07 |  | 0.65 | 0.07 |  | 0.65 | 0.05 |
| DOS+DVH | ET |  | 0.69 | 0.51-0.79 | 0.07 |  | 0.62 | 0.08 |  | 0.61 | 0.14 |  | 0.62 | 0.08 |  | 0.57 | 0.19 |
| DOS+DVH | GTB |  | 0.68 | 0.56-0.83 | 0.07 |  | 0.64 | 0.08 |  | 0.67 | 0.08 |  | 0.66 | 0.08 |  | 0.66 | 0.07 |
| DOS+DVH | RF |  | 0.71 | 0.56-0.83 | 0.07 |  | 0.65 | 0.07 |  | 0.69 | 0.08 |  | 0.67 | 0.07 |  | 0.67 | 0.07 |
| DOS+DVH | NB |  | 0.74 | 0.66-0.80 | 0.04 |  | 0.68 | 0.05 |  | 0.69 | 0.05 |  | 0.69 | 0.06 |  | 0.66 | 0.07 |
| DOS+RTP | SVM |  | 0.69 | 0.54-0.81 | 0.06 |  | 0.60 | 0.05 |  | 0.64 | 0.07 |  | 0.61 | 0.05 |  | 0.59 | 0.10 |
| DOS+RTP | KNN |  | 0.75 | 0.60-0.89 | 0.08 |  | 0.66 | 0.07 |  | 0.70 | 0.07 |  | 0.67 | 0.07 |  | 0.67 | 0.08 |
| DOS+RTP | LR |  | 0.55 | 0.33-0.78 | 0.13 |  | 0.51 | 0.11 |  | 0.53 | 0.13 |  | 0.52 | 0.12 |  | 0.52 | 0.13 |
| DOS+RTP | ET |  | 0.75 | 0.58-0.82 | 0.06 |  | 0.66 | 0.07 |  | 0.71 | 0.09 |  | 0.68 | 0.07 |  | 0.67 | 0.10 |
| DOS+RTP | GTB |  | 0.70 | 0.52-0.85 | 0.09 |  | 0.63 | 0.09 |  | 0.68 | 0.10 |  | 0.65 | 0.09 |  | 0.65 | 0.09 |
| DOS+RTP | RF |  | 0.69 | 0.52-0.81 | 0.08 |  | 0.62 | 0.08 |  | 0.66 | 0.09 |  | 0.64 | 0.08 |  | 0.64 | 0.09 |
| DOS+RTP | NB |  | 0.69 | 0.57-0.77 | 0.05 |  | 0.59 | 0.05 |  | 0.64 | 0.08 |  | 0.61 | 0.06 |  | 0.61 | 0.09 |
| DOS+DVH+RTP | SVM |  | 0.75 | 0.64-0.83 | 0.05 |  | 0.64 | 0.08 |  | 0.70 | 0.11 |  | 0.64 | 0.06 |  | 0.59 | 0.12 |
| DOS+DVH+RTP | KNN |  | 0.66 | 0.51-0.81 | 0.08 |  | 0.60 | 0.08 |  | 0.63 | 0.12 |  | 0.61 | 0.08 |  | 0.55 | 0.14 |
| DOS+DVH+RTP | LR |  | 0.82 | 0.74-0.89 | 0.05 |  | 0.65 | 0.06 |  | 0.69 | 0.05 |  | 0.67 | 0.06 |  | 0.68 | 0.05 |
| DOS+DVH+RTP | ET |  | **0.83** | 0.71-0.90 | 0.05 |  | **0.70** | 0.07 |  | **0.74** | 0.06 |  | **0.72** | 0.06 |  | **0.72** | 0.06 |
| DOS+DVH+RTP | GTB |  | 0.75 | 0.55-0.88 | 0.08 |  | 0.65 | 0.07 |  | 0.69 | 0.08 |  | 0.67 | 0.07 |  | 0.67 | 0.08 |
| DOS+DVH+RTP | RF |  | 0.71 | 0.53-0.84 | 0.08 |  | 0.63 | 0.08 |  | 0.66 | 0.09 |  | 0.64 | 0.08 |  | 0.63 | 0.09 |
| DOS+DVH+RTP | NB |  | 0.78 | 0.69-0.85 | 0.04 |  | 0.65 | 0.05 |  | 0.68 | 0.06 |  | 0.66 | 0.05 |  | 0.67 | 0.06 |

| Supplementary Table 2. Overview of significant features indicating AST 2+ with a Two-Tailed t-Test. | | | | | | | | | |
| --- | --- | --- | --- | --- | --- | --- | --- | --- | --- |
| Feature | |  | CTCAE <2 | |  | CTCAE≥2 | |  | p_value |
| Class | Name |  | Mean | STD |  | Mean | STD |  | 0.05 |
| First order | Range | | 51.66 | 4.95 |  | 59.09 | 5.15 |  | 0.000 |
|  | Skewness | | -1.16 | 0.37 |  | -0.78 | 0.83 |  | 0.048 |
|  | Uniformity | | 0.05 | 0.02 |  | 0.03 | 0.01 |  | 0.018 |
| GLCM | Contrast | | 9.64 | 4.37 |  | 24.83 | 24.18 |  | 0.007 |
|  | Correlation | | 0.96 | 0.03 |  | 0.92 | 0.07 |  | 0.029 |
|  | Difference Average | | 2.25 | 0.68 |  | 3.36 | 1.76 |  | 0.008 |
|  | Difference Entropy | | 2.59 | 0.41 |  | 3.03 | 0.63 |  | 0.006 |
|  | Difference Variance | | 3.54 | 1.35 |  | 9.35 | 8.93 |  | 0.005 |
|  | Idmn |  | 1.00 | 0.00 |  | 0.99 | 0.01 |  | 0.019 |
|  | Imc2 |  | 0.98 | 0.01 |  | 0.97 | 0.03 |  | 0.022 |
|  | Inverse Variance | | 0.32 | 0.04 |  | 0.28 | 0.08 |  | 0.021 |
|  | Joint Entropy | | 7.92 | 0.78 |  | 8.73 | 0.90 |  | 0.001 |
|  | MCC |  | 0.99 | 0.01 |  | 0.95 | 0.07 |  | 0.008 |
|  | Sum Entropy | | 5.86 | 0.37 |  | 6.20 | 0.33 |  | 0.001 |
| GLDM | Dependence Entropy | | 7.87 | 0.39 |  | 8.12 | 0.48 |  | 0.035 |
|  | Small Dependence Emphasis | | 0.17 | 0.07 |  | 0.21 | 0.09 |  | 0.034 |
|  | Small Dependence Low Gray Level Emphasis | | 0.00 | 0.00 |  | 0.00 | 0.00 |  | 0.011 |
| GLRLM | Gray Level Non Uniformity Normalized | | 0.04 | 0.01 |  | 0.03 | 0.01 |  | 0.004 |
|  | Run Entropy | | 5.58 | 0.32 |  | 5.82 | 0.37 |  | 0.013 |
|  | Short Run Low Gray Level Emphasis | | 0.01 | 0.01 |  | 0.02 | 0.02 |  | 0.031 |
| GLSZM | Gray Level Non Uniformity Normalized | | 0.04 | 0.01 |  | 0.04 | 0.01 |  | 0.037 |
|  | Gray Level Variance | | 63.46 | 32.03 |  | 97.92 | 59.50 |  | 0.018 |
|  | Low Gray Level Zone Emphasis | | 0.00 | 0.00 |  | 0.01 | 0.01 |  | 0.014 |
|  | Size Zone Non Uniformity | | 7263.49 | 4706.55 |  | 12143.12 | 7767.23 |  | 0.007 |
|  | Small Area Low Gray Level Emphasis | | 0.00 | 0.00 |  | 0.00 | 0.00 |  | 0.013 |
|  | Zone Percentage | | 0.17 | 0.08 |  | 0.23 | 0.11 |  | 0.029 |
| NGTDM | Complexity | | 1806.57 | 794.03 |  | 3275.07 | 2033.21 |  | 0.003 |
|  | Contrast | | 0.14 | 0.06 |  | 0.19 | 0.09 |  | 0.027 |
| DVH | V_45 Gy_ (cc) |  | 27.66 | 25.33 |  | 43.57 | 26.61 |  | 0.032 |
|  | V_50 Gy_ (cc) |  | 7.04 | 8.72 |  | 17.20 | 16.07 |  | 0.010 |
|  | V_55 Gy_ (cc) |  | 1.22 | 3.17 |  | 5.11 | 6.20 |  | 0.010 |
|  | V_60 Gy_ (cc) |  | 0.06 | 0.20 |  | 0.77 | 1.55 |  | 0.039 |
|  | D _max_ |  | 52.76 | 5.48 |  | 57.93 | 4.61 |  | 0.001 |
|  | V_50 Gy_ (%) |  | 5.23 | 6.16 |  | 10.85 | 11.78 |  | 0.047 |
|  | V_55 Gy_ (%) |  | 0.83 | 1.99 |  | 3.21 | 5.13 |  | 0.045 |
| PTR | Fraction schedule | | 0.52 | 0.51 |  | 0.96 | 0.21 |  | 0.000 |
|  |  |  |  |  |  |  |  |  |  |

**
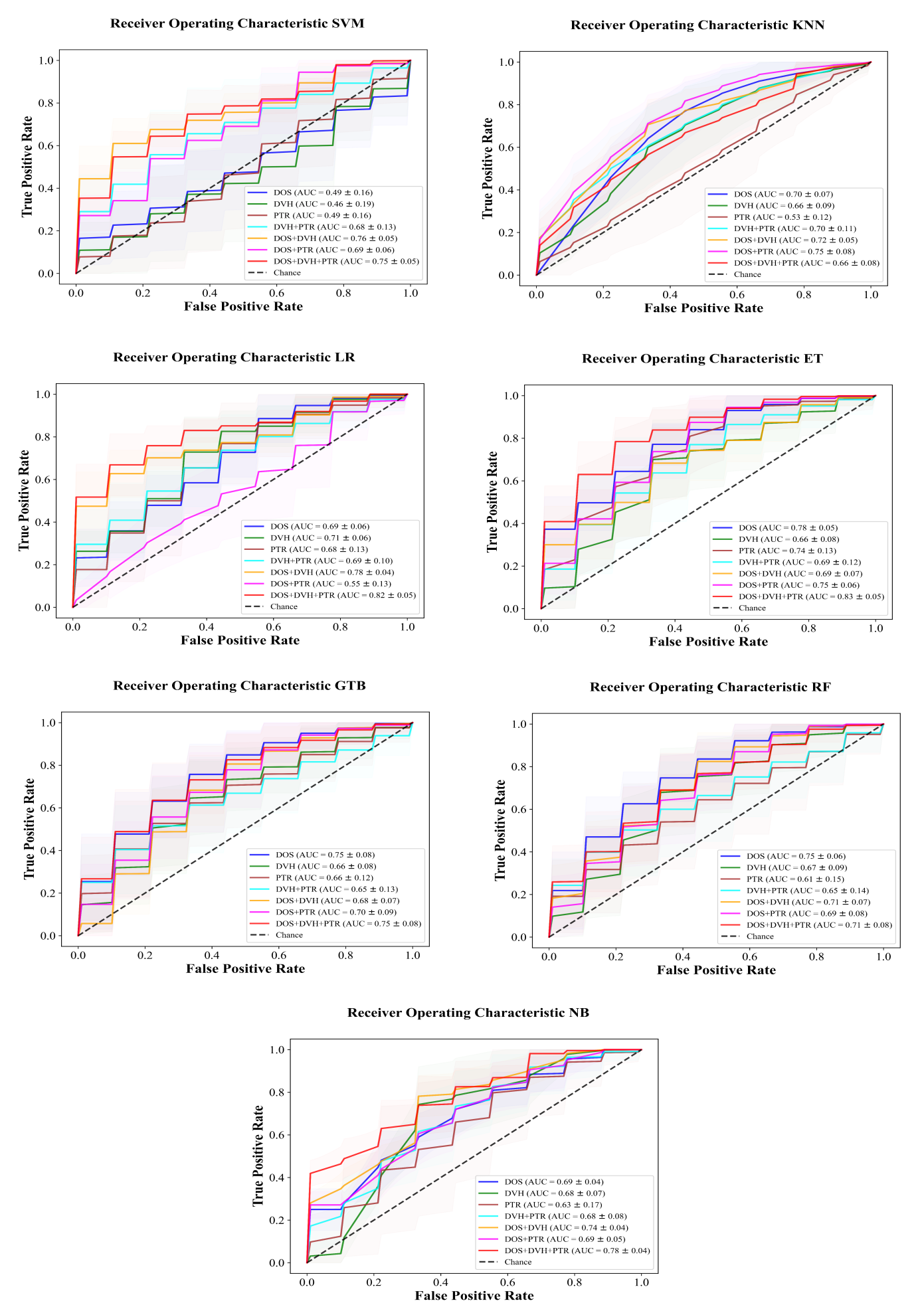
** Supplementary Fig 1. Comparing Model Performance in Testing Cohorts via ROC Curves. The blue, green, brown, cyan, orange, pink, and red lines represent the results of DOS, DVH, PTR, DVH+PTR, DOS+DVH, DOS+PTR and, DOS+DVH+PTR ML models.

Supplementary File 1. The hyper-parameters for the seven classifiers

# Define the random search parameters for each model

params = {

'Support Vector Machine': {'C' : np.logspace(-5,10,1500, base=2),

                                     'gamma': np.logspace(-15,3,180, base=2)},

          'k-Nearest Neighbors': {'n_neighbors' : [1,2,3,4,5,6,7,8,9,10,11,12,13,14,15],

                                  'p': [1,2]},

          'Logistic Regression': {'penalty' : ['l1', 'l2', 'elasticnet', 'none'],

                                  'l1_ratio': [0,1],  'C' : np.logspace(-5,10,100, base=2),

                                  'solver' : ['lbfgs','newton-cg','liblinear','sag','saga'],

                                  'max_iter' : [100, 1000,2500, 5000]  },

          'Extra-Trees': {'n_estimators' : [90, 230],

                          'criterion': ["gini", "entropy"],

                          'max_features'  :np.arange(0.05,1.05,0.05),

                          'min_samples_split': np.arange(2,21,1),

                          'min_samples_leaf': np.arange(1,21,1)},

          'Gradient Tree Boosting':{'n_estimators' : [200, 2000],

                                    'learning_rate' : np.logspace(-7,-1,60, base=2),

                                    'max_depth': np.arange(1,7,1),

                                    'subsample': np.arange(0.6,1.05,0.05)},

          'Random Forest':{'bootstrap': [True, False],

                           'max_depth': [ 5, 10, 20, 30, 40, 50, 60, 70, 80, 90, 100, None],

                           'max_features': ['auto', 'sqrt', None],

                           'min_samples_leaf': [1, 2, 4],

                           'min_samples_split': [2, 5, 10],

                           'n_estimators': np.arange(5,205,5),

                           'max_leaf_nodes': [3, 6, 9,None ]},

          'Naive Bayes':{'alpha': [0.1, 0.5, 1.0, 1.5, 2.0],

                         'binarize': [0.0, 0.1, 0.2, 0.3, 0.4, None]}

}
